# Late aortic lymphocele and residual ovary syndrome after gynecological surgery

**DOI:** 10.1186/1477-7819-5-146

**Published:** 2007-12-28

**Authors:** Maria Pastore, Natalina Manci, Claudia Marchetti, Francesca Esposito, Marialetizia Iuliano, Lucia Manganaro, Pierluigi Benedetti Panici

**Affiliations:** 1 Dept of Obstetrics and Gynecology, "La Sapienza" University, Rome, Italy; 2 Dept of Radiology, "La Sapienza" University, Rome, Italy

## Abstract

**Background:**

Gynecological surgery, as radical hysterectomy or pelvic and aortic lymphadenectomy, accounts for more than 50% of iatrogenic injuries. In premenopausal women, an hysterectomy with ovarian sparing and concomitant lateral ovarian transposition is frequently performed. However, the fate of the retained ovary is complicated by the residual ovarian syndrome (ROS) and one of the most common postoperative complications of the lymphadenectomy procedure is the lymphocele, with an average incidence of 22–48.5%. The differential diagnosis of a postoperative fluid collection includes, in addition to a lymphocele, urinoma, hematoma, seroma or abscess and the computed tomography (CT) findings alone is not enough.

**Case presentation:**

We describe a patient, affected by ROS concomitant with a asymptomatic lymphocele, initially confused with an aortic lymph nodes relapse, after abdominal radical hysterectomy. The patient was subjected to a surgical approach, included a diagnostic open laparoscopy and laparotomy with sovraombelico-pubic incision, wide opening of the pelvic peritoneum and retroperitoneum. Examination of the mass revealed, macroscopically, a ovary with multiloculated cystic masses filled with clear or yellow serous fluid and the layers were composed by flat or cuboidal mesothelial cells.

**Conclusion:**

The tribute of this case illustrates the atypical appearance with uncertain aetiology after complex imaging. Gynecologist and radiologist should acquaint with the appearance of fluid collection (urinoma, lymphocele, seroma, hematoma, abscess) in gynecologic oncology follow-up to properly differentiated from tumor recurrence.

## Background

Gynecological surgery, as radical hysterectomy or pelvic and aortic lymphadenectomy, accounts for more than 50% of iatrogenic injuries [1,2], such as urinoma, lymphocele, haematoma, seroma or abscess [3-5]. Furthermore, women of reproductive age having an hysterectomy because of benign or malignant disease require the gynecologist to decide whether to preserve or to remove the gonads. Beyond the age of 40 and especially over 45 years, many surgeons perform the removal of the ovaries. On converse, the preservation of one or both ovaries at the time of hysterectomy (retained ovary) consents to preserve the important role in steroid metabolism even after the menopause, but the residual ovary syndrome (ROS) results in the 2–3% of women [6-8].

We describe a case of ROS concomitant with a pregress asymptomatic lymphocele, in a 37-year-old woman who developed an abdominopelvic mass, initially confused with a recurrence in the aortic lymph nodes, 12 years after abdominal radical hysterectomy for cervical cancer.

## Case presentation

A 37-year-old woman was admitted to our Institute with suspected an abdominopelvic mass, without univocal interpretation, considering the peculiarity of the onset and of the radiological appearance.

Twelve years earlier, the patient was treated with radical abdominal hysterectomy. Whertheim-Meigs radical hysterectomy, systematic pelvic and aortic lymphadenectomy with conservation and lateral transposition of the right ovary for stage IB1 cervical cancer. One of 47 pelvic lymph nodes removed was positive. Adjuvant external radiotherapy was administered for a total of 4500 cGy. At the end of treatment, the patient developed asymptomatic aortic lymphocele, measuring 4 cm in diameter and it was untreated. The follow-up was negative until December 2006 when the patient exhibited dyspareunia, lower abdominal and back pain. The first examinations carried out, ultrasonography and computed tomography (CT) showed an irregular loculated cystic mass measuring 8 × 7 cm, located in aortic area. The serum level of CA125 was found increased to 89 U/mL and there was a slow and progressive elevation.

**Figure 1:**
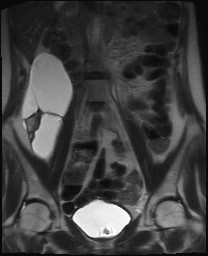
Coronal T2-weighted image shows multiloculated nature of cystic retroperitoneal mass. Surgical pathology confirmed multicistic ovarian tissue with lymphocele adherent to it.

**Figure 2:**
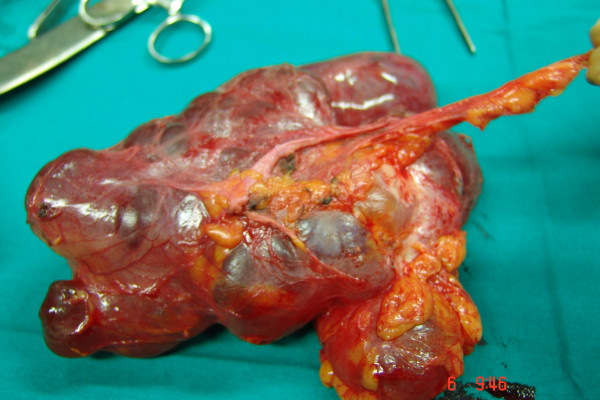
Surgical specimen of right ovary. Ovarian pedicle adherent to aortic lymphocele are both visible.

**Figure 3:**
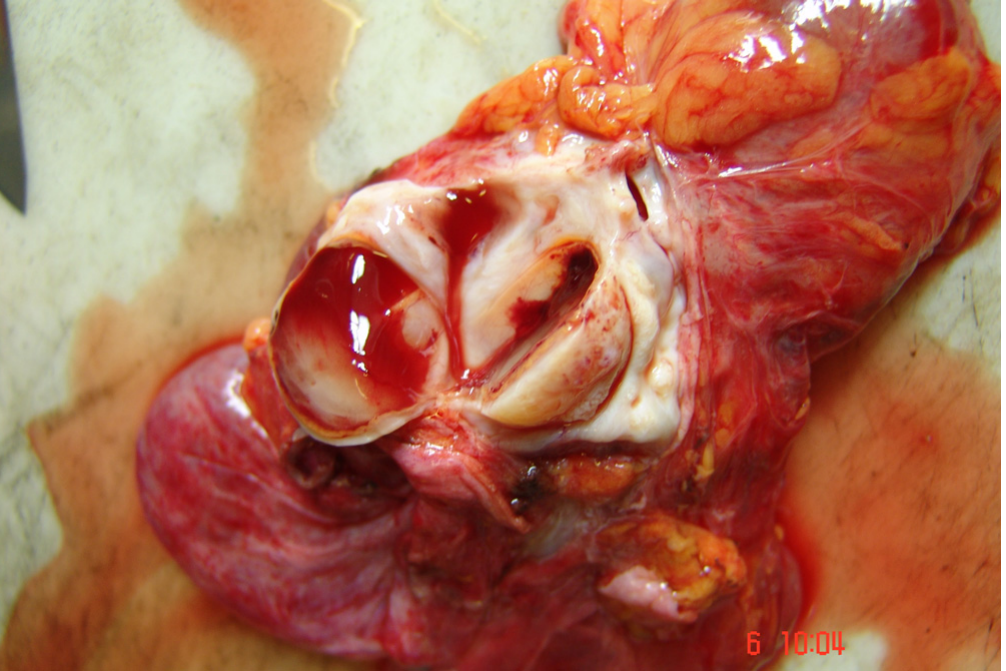
Multifollicolar ovarian tissue inside retroperitoneal mass mimicking nodal aortic recurrence.

These symptoms worsened and the first diagnosis was confirmed by subsequent several imaging studies. In March 2007, the patient underwent to further abdominal and pelvic CT scan that explained a pelvic mass with complex features; pelvic MRI confirmed a cystic pelvic mass of 18 × 8 cm size but with a solid component (figure 1). About the radiological appearance of a complex cystic mass, strongly evocative for a tumor recurrence, the oncological radiologist equip dissuaded from any diagnostic approaches, such as fine-niddle aspiration, sclerotherapy, catheter drainage. For all these reasons, the patient was subjected to a surgical approach, included a diagnostic open laparoscopy and laparotomy with sovraombelico-pubic incision, wide opening of the pelvic peritoneum and retroperitoneum. Because of adhesions and distorted anatomy, the dislocated ureters were at risk during surgery and a retrograde dissection was necessary. Frozen section examination of the mass revealed, macroscopically, a ovary with multiloculated cystic masses filled with clear or yellow serous fluid and the layers were composed by flat or cuboidal mesothelial cells. It was observed a lymphocele adherent to it (figure 2, figure 3). The patient made an uneventful recovery and was discharged 4 days later. After surgery, symptoms disappeared and she was well at her two weeks postoperative check-up and up to now.

## Discussion

Lymphadenectomy plays an important role in the management of gynecological malignancies for the assessment of lymph node status. In premenopausal women, an hysterectomy with ovarian sparing and concomitant lateral ovarian transposition is frequently performed.

However, the fate of the retained ovary could be complicated by the residual ovarian syndrome (ROS): it consists of recurrent or chronic pelvic pain (71–77%), disturbance of the urinary tract (33%), dyspareunia (67%), pelvic mass on bimanual examination (14–25%), as a single or a cluster of symptoms. The interval between hysterectomy and the appearance of symptoms surgery for the residual adnexal disease syndrome range from 4 months to 26 years. Over 50% of the women presented within 5 years, and 75% within 10 years. It is estimated that at least 2.85% of patients will develop ROS and require surgery for it following hysterectomy [9,10]. In literature, it is also described an increased incidence of functional ovarian cysts and peritoneal inclusion cysts, partially accounting the above mentioned symptoms.

All at once, one of the most common postoperative complications of the lymphadenectomy procedure is the lymphocele, with an average incidence of 22–48.5% [4]. Usually, it became detectable within 3–8 weeks after surgery and the symptomatology depends by the size, the locations and the possible sovrainfection. The differential diagnosis of a postoperative fluid collection includes, in addition to the lymphocele, urinoma, hematoma, seroma or abscess [11]. About abdominal hematoma and abscess the diagnosis seems easier. The urinoma is an encapsulated collection of urine that forms from urine leakage and its radiological appearance is of a soft tissue mass mistaken with lymphocele. Urinary symptoms, percutaneous fluid chemistries analysis or delayed CT scanning demonstrating ureteral extravasation is crucial to differentiate urinomas rather than lymphoceles.

In this case, the presence of a small, asymptomatic lymphocele was diagnosed 6 weeks after surgical procedure by CT scan. Considering that most of the small lymphocysts regress spontaneously, a conservative approach was opted. Remarkably, in this report the lymphocyst preserved the same aspects, either for the size and the location, until 12 years later, when a CT showed irregular cystic pelvic mass, mimicking malignant recurrent ovarian/lymphonodal lesion. Although late relapse for cervical cancer after the fifth years is very rare, estimated about 04–7.5%, one third of it had lymph node metastasis and the CT scan is helpful for early diagnosis [12]. This hypothesis was also corroborated by the increase of the tumor marker CA 125. The patient was finally subjected to surgery because, according with Kim *et al*. [3], lymphoceles occurring later than 1 year should be subjected to a more thorough diagnostic investigation in order to exclude a recurrent disease. In our experience there have been no previously documented cases in which such final diagnosis was registered.

## Conclusion

This case emphasizes that the gynecologist and radiologist should acquaint with the appearance of transposed ovaries and their abnormalities, to properly differentiated from other possible entities, such as peritoneal metastases, urinomas, seroma, hematoma, abscess, cystic neoplasm; the report also strongly supports the evidence that more close radiological examinations in this setting of patients are required in order to promptly exclude tumor recurrence.

## Competing interests

The author(s) declare that they have no competing interests.

## Authors' contributions

**MP **Conceived of the study. Carried out the design of the study, participated in the sequence alignment and drafted the manuscript. **NM **Participated in the study's design and coordination. Gave final approval of the version to be published. **CM**. Carried out the analysis and interpretation of data. Helped to draft the manuscript. **FE **Participated in the study's design and coordination. Performed the statistical analysis. **MI **Helped to draft the manuscript. Participated in the design. **LM **Carried out the acquisition of data. Participated in the design. **PBP **Gave final approval of the version to be published and revised the manuscript.

All authors read and approved the manuscript.
